# Cytotoxic, Genotoxic, and Polymorphism Effects on *Vanilla planifolia* Jacks ex Andrews after Long-Term Exposure to Argovit^®^ Silver Nanoparticles

**DOI:** 10.3390/nano8100754

**Published:** 2018-09-25

**Authors:** Jericó Jabín Bello-Bello, José Luis Spinoso-Castillo, Samantha Arano-Avalos, Eduardo Martínez-Estrada, María Evarista Arellano-García, Alexey Pestryakov, Yanis Toledano-Magaña, Juan Carlos García-Ramos, Nina Bogdanchikova

**Affiliations:** 1 Conacyt-Colegio de Postgraduados Campus Córdoba, Carretera Córdoba Veracruz, Amatlán de los Reyes Km 348, Veracruz 94946, Mexico; 2 Colegio de Postgraduados Campus Córdoba, Carretera Córdoba Veracruz, Amatlán de los Reyes Km 348, Veracruz 94946, Mexico; jlspinoso@gmail.com (J.L.S.-C.); samy.a.avalos@gmail.com (S.A.-A.); eduagrom@hotmail.com (E.M.-E.); 3 Facultad de Ciencias, Universidad Autónoma de Baja California, Carretera Transpeninsular Tijuana 3917, Ensenada 22860, Mexico; evarista.arellano@uabc.edu.mx; 4 Research School of Chemical and Biomedical Technologies, Tomsk Polytechnic University, Lenin Avenue 30, Tomsk 634050, Russia; pestryakov2005@yandex.ru; 5 Conacyt-Universidad Nacional Autónoma de México-Centro de Nanociencias y Nanotecnología, Carretera Tijuana-Ensenada Km 107, Ensenada 22860, Mexico; yanistoledano@cnyn.unam.mx; 6 Centro de Nanociencias y Nanotecnología, Universidad Nacional Autónoma de México, Carretera Tijuana-Ensenada Km 107, Ensenada 22860, México; nina@cnyn.unam.mx

**Keywords:** silver nanoparticles, *Vanilla planifolia*, growth promotion, cytotoxicity, genotoxicity, polymorphism induction, safe nanoparticles

## Abstract

Worldwide demands of *Vanilla planifolia* lead to finding new options to produce large-scale and contaminant-free crops. Particularly, the Mexican Government has classified *Vanilla planifolia* at risk and it subject to protection programs since wild species are in danger of extinction and no more than 30 clones have been found. Nanotechnology could help to solve both demands and genetic variability, but toxicological concerns must be solved. In this work, we present the first study of the cytotoxic and genotoxic effects promoted by AgNPs in *Vanilla planifolia* plantlets after a very long exposure time of six weeks. Our results show that *Vanilla planifolia* plantlets growth with doses of 25 and 50 mg/L is favored with a small decrease in the mitotic index. A dose-dependency in the frequency of cells with chromosomal aberrations and micronuclei was found. However, genotoxic effects could be considered as minimum due to with the highest concentration employed (200 mg/L), the total percentage of chromatic aberrations is lower than 5% with only three micronuclei in 3000 cells, despite the long-time exposure to AgNP. Therefore, 25 and 50 mg/L (1.5 and 3 mg/L of metallic silver) were identified as safe concentrations for *Vanilla planifolia* growth on in vitro conditions. Exposure of plantlets to AgNPs increase the polymorphism registered by inter-simple sequence repeat method (ISSR), which could be useful to promote the genetic variability of this species.

## 1. Introduction

*Vanilla planifolia* is the source of vanillin, a widely used raw material of the pharmaceutical, food, and cosmetic industry. This poses some challenges, like the large-scale and contaminant-free cultivation of this species to meet the growing requirements of the industry [1]. Usually, it is propagated asexually by cuttings, which does not guarantee the health of the new plantations. An alternative to avoid these difficulties emerges with the combination of nanotechnology and plant tissue culture (PTC), which is useful for manipulation, conservation, and regeneration of plants [2,3].

Recently, the application of silver nanoparticles (AgNPs) in PTC has been raised due to their capacity to eliminate the microorganisms that affect crops [4,5]. Particularly, explant disinfection protocols during in vitro establishment [6,7] with AgNPs produce very good results. However, major concerns appear regarding toxicological and environmental effects which must be solved before AgNPs’ indiscriminate and spreading use [8,9]. As adverse effects manifest strongly depending on plant sensitivity, the identification of safety windows for AgNPs use in each species is highly recommended. Some examples of problems produced by the use of higher amounts of AgNPs are the slow growth of vanilla, poplars, and *Arabidopsis*, or the decrease of mung bean seed germination. [1,10,11].

Most of the toxicological effects produced by exposure of plants to AgNPs considers physiological endpoints, such as number and length of shoots and roots or biochemical markers as reactive oxygen species (ROS) concentration. However, much more effort must be designated in the evaluation of genetic endpoints, monitoring chromosomic aberrations, the appearance of micronuclei, DNA damage, chromatid exchanges, among others [12]. To achieve this goal, several DNA-based techniques like Inter-Simple Sequence Repeat (ISSR) [13] or amplified fragment length polymorphism (AFLP) [14] are employed, with the advantage that both techniques also help in the identification and quantification of polymorphism.

Last year, our group reported the efficacy of the AgNPs’ commercial formulation to eliminate contaminants from *Vanilla planifolia* plantlets through a temporary immersion system that is going to be used on in vitro regeneration procedures. In this work 30 days of exposure to concentrations higher than 50 mg/L generates adverse effects in the development of plantlets, mainly by the high increase of ROS induced by the nanoparticles that overwhelmed the antioxidant response of the plant. Continuing with the current work will help to enhance the knowledge regarding beneficial and adverse effects of the use of AgNPs in plants.

In this work, we studied the cytotoxic and genotoxic effects on *Vanilla planifolia* plantlets exposed to different concentrations of AgNPs for six weeks, two weeks more than the past study. We evaluate the mitotic index of treated plantlets compared to negative controls and the potential genotoxic effect determining the frequency of chromatic aberrations appearance (bridges, budges, chromosomal fragments and micronuclei) on plantlets exposed to this AgNPs formulation. Furthermore, for the safe concentrations, we explore the capacity of this AgNPs formulation to improve the genetic variability through the induction of polymorphism. As far as we know, this is the first study that monitors the genotoxicity and somaclonal variations on *Vanilla planifolia* Jacks. ex Andrews under in vitro conditions and with a long-term exposure to the nanomaterial.

## 2. Materials and Methods

### 2.1. In Vitro Establishment and Culture Conditions

Stems of 20 cm length were cut from young *V. planifolia* plants kept under greenhouse conditions. The leaves were removed, and 2 cm length nodal segments were cut off for use as explants. These were washed with a toothbrush and a solution prepared with 1 L tap water and 2 drops of Tween-20 (Sigma-Aldrich Chemical Company, St. Louis, MO, USA) for 45 min. The explants were transferred to a laminar flow hood and immersed for 30 s in 70% ethanol (*v*/*v*) solution, then rinsed three times with sterile distilled water. The explants were immersed in sodium hypochlorite solutions of final concentrations 0.6 and 0.3% (*v*/*v*) for 10 and 5 min, respectively; after that were rinsed three times with sterile distilled water. Explants were cultured in 2.2 × 15 cm test tubes with 15 mL MS medium supplemented with 3 g/L of sucrose without growth regulators. Culture medium pH was adjusted with 0.1 N sodium hydroxide until pH = 5.8. 0.25% (*w*/*v*), Phytagel (Sigma Chemical Company, St. Louis, MO, USA) was added as a gelling agent, and then it was autoclaved for 15 min at 120 °C. The explants were incubated at 24 ± 2 °C, 16 h light photoperiod with 40 µmol m^−2^·s^−1^. After two subcultures of four weeks each, 2 cm length shoots were used for different treatments with AgNPs.

### 2.2. Silver Nanoparticles (AgNPs)

Commercial AgNPs formulation, Argovit^®^, was obtained from Scientific-Production Centre Vector-Vita Ltd., Novosibirsk, Russia. Argovit^®^ is a water suspension of AgNPs with an average size of 38 ± 15 nm coated with polyvinylpyrrolidone (PVP). The supplier’s specifications indicate a metallic content of 12 mg/mL with 188 mg/mL of coating agent to generate a 20% AgNPs (200 mg/mL) suspension. AgNPs characterization was performed by Transmission Electron Microscopy (TEM, JEOL JEM-2010, Tokyo, Japan) and silver content determined by Inductively coupled plasma-optical emission spectroscopy (ICP-OES, Varian, Palo Alto, CA, USA) before using. Z-potential was determined in a Malvern Instruments Zetasizer Nano NS model DTS 1060 (Malvern Instruments, Worcestershire, UK) in triplicate.

### 2.3. Effect of AgNPs on In Vitro Elongation and Rooting of V. planifolia

Each experimental tube (2.2 × 15 cm test tubes) contains two shoots of 2 cm length cultured with 20 mL MS medium without growth regulators, supplemented with 30 g/L of sucrose and the corresponding concentrations of AgNPs (0, 25, 50, 100, and 200 mg/L). Ten test tubes were used per treatment. Culture medium pH adjustment, autoclaving and culture conditions were the same as described above. After six weeks of culture, shoot length, roots number and length, and the number of leaves was evaluated for all treatments.

### 2.4. Genotoxic Effect of AgNPs on V. planifolia

To determine the possible genotoxic effect on *Vanilla planifolia* plantlets exposed to different treatments of AgNPs, the root tip chromosomal aberration assay of *Vicia faba* of the International Program on Chemical Safety (IPCS, WHO) [15] was used because of its simplicity, quickness, and inexpensiveness with respect to the procedures for the obtainment of reliable results.

#### 2.4.1. Fixation and Staining of Root Tips

Roots’ tips were fixed in a freshly prepared fixative solution containing three parts methanol and one-part of glacial acetic acid, this solution was kept at 4 °C until its use. For preparing the root tips smears, they were removed from the refrigerator and transferred to room temperature in distilled water for 5 min. The root tips were then hydrolyzed with 1 N HCl at 60 °C for 6–7 min. After hydrolysis, the root tips were thoroughly washed with water several times and then stained with aceto-orcein stain. Aceto-orcein stain is prepared adding 1 g of Orcein (Sigma-Aldrich, St. Louis, MO, USA) powder to 55 mL of boiling acetic acid at 45% in constant stirring. Once cool, the solution was adjusted to 100 mL with distilled water. The final solution is filtered and ready to use. When staining was completed, (after 45–60 min) the root-tips were transferred to clean slides and the darkly stained tips containing the meristem were separated from the rest of the roots. Squash preparations were produced in 45% acetic acid.

#### 2.4.2. Scoring of Slides

In *V. planifolia* chromosome aberration assay, slides were scored for chromatid and chromosome aberrations only in metaphase. Six hundred cells in metaphases per root-tip and a total of 3000 cells were used for each treatment to obtain the total number of chromosomal aberrations. The mitotic index was obtained by counting the number of mitotic cells in 3000 cells per treatment using an Olympus microscope (Shinjuku-ku, Tokyo, Japan). The mitotic index was calculated as the ratio of the number of dividing cells to the total number of cells, multiplied by 100. The aberrations scored were chromatid breaks, lagging chromosomes, binucleated cells, and micronucleus.

### 2.5. Effect of AgNPs on Somaclonal Variation

#### 2.5.1. DNA Isolation

Leaf samples from five randomly-selected shoots per treatment were used for DNA genomic extraction. The extraction was accomplished according to the CTAB (cetyl trimethylammonium bromide) method, described by Stewart and Via [16]. The integrity of the extracted DNA was verified in 1% agarose gel stained with 10 mg/L ethidium bromide. Quantity and purity of the DNA were evaluated by spectrophotometry (Genesys 10S UV-VIS, Thermo Scientific, Vernon Hills, IL, USA).

#### 2.5.2. ISSR-PCR Analysis

Thirty primers were tested to screen the DNA polymorphism in *V. planifolia*, from which the nine primers showing best quality of amplification profile were selected (Table 1). The reactions were carried out in a final volume of 25 μL containing 50 ng of DNA template, 1X PCR reaction buffer, 2.5 mM of MgCl_2_, 0.2 mM of dNTPS, 0.2 μM of primer and 1 U of Taq DNA polymerase (Sigma-Aldrich Chemical Company, St. Louis, MO, USA). DNA amplification was performed in a MaxyGene thermocycler (Axygen, Tewksbury, MA, USA) using the following cycling program: one cycle at 94 °C for 4 min; 35 cycles at 94 °C for 50 s, 45–62 °C (according to the primer) for 50 s and 72 °C for 90 s; and a final extension at 72 °C for 10 min. The amplification products were separated by electrophoresis on 3% agarose gels at 90 V for 90 min and stained with 10 mg/L ethidium bromide. A DNA ladder (50–10,000 bp, DirectLoad Wide Range DNA marker, Sigma-Aldrich Chemical Company, St. Louis, MO, USA) was used as a molecular weight marker. The gels were photographed under UV light, using a Gel Doc-It Imager photo-documentation system (UVP, Upland, CA, USA). For each treatment, the polymorphism (%) was calculated.

### 2.6. Experimental Design and Statistical Analysis

The experiment was conducted using a completely randomized design consisting of five treatments with three replications; each replication included ten test tubes. For genotoxic effect determinations, three samples were used. For each sample, 600 cells in metaphase were analyzed per root-tip and five root-tips per treatment. For all variables, except molecular data, an analysis of variance and Tukey’s comparison of means test (*p* ≤ 0.05) were performed using SPSS statistical software (Version 11.5 for Windows Inc., Chicago, IL, USA).

## 3. Results and Discussion

### 3.1. Characterization of AgNPs

**Figure 1:**
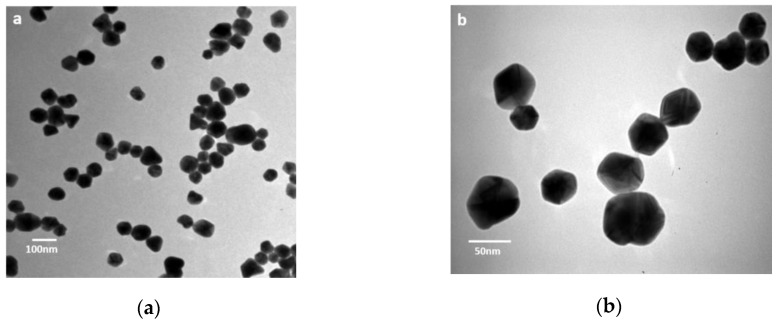
TEM image of AgNPs batch used in this work using different magnifications. (**a**) bar = 100 nm, and (**b**) bar = 50 nm.

The batch of the commercial AgNPs formulation (Argovit^®^) employed in this work was completely characterized, and physicochemical characteristics are presented in Table 2. The AgNPs characterized by TEM showed a spherical form (form factor 0.82) with a roundness of 0.88 (Figure 1). Size interval of silver nanoparticles is in the range 1–80 nm. The analysis of the AgNPs’ dimensions showed average diameters of 38 ± 15 nm. Silver content quantification determined by ICP-OES has shown a concentration of 12 mg/mL). Other physicochemical parameters agree with those reported by the provider.

### 3.2. Effects of AgNPs on Vanilla planifolia Physiological Parameters

**Figure 2:**
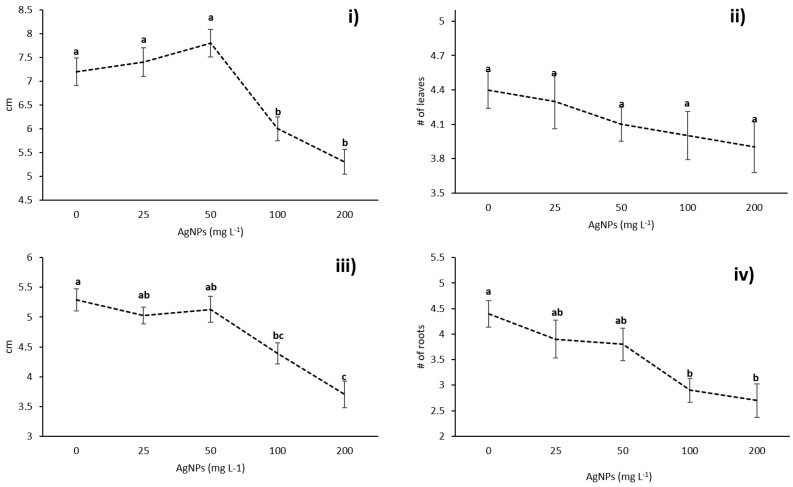
Effect of AgNPs on in vitro growth of *V. planifolia* after six weeks of culture. (**i**) Shoot length, (**ii**) number of leaves, (**iii**) root length, and (**iv**) number of roots. Different letters denote statistically significant differences according to Tukey’s test (*p* ≤ 0.05).

Administration of silver nanoparticles to *Vanilla planifolia* in vitro show a dose-dependent effect in different growth parameters such as shoot length and the number and length of roots. The shoot length, the number of leaves, and the number and length of the roots of plants exposed to 25 and 50 mg/L of AgNPs (1.5 and 3.0 mg/L of metallic silver) show no significant differences compared with the untreated plants used as a negative control group. On the other hand, the plants exposed to the higher concentrations of 100 and 200 mg/L of AgNPs (6 and 12 mg/L of metallic silver, respectively) show a minor number of roots with a decrease in its length and a decrease in the length of the shoots, but no difference regarding the number of leaves (Figure 2).

**Figure 3:**
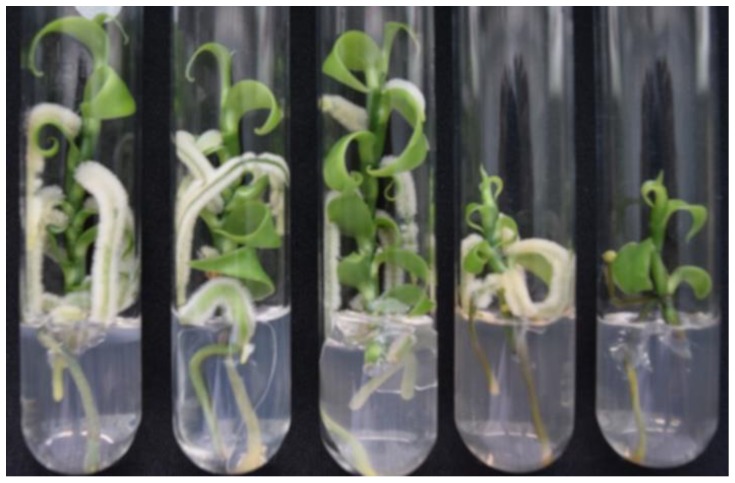
Effect of AgNPs on in vitro elongation and rooting of *V. planifolia* after six weeks of in vitro culture. From left to right 0, 25, 50, 100, and 200 mg/L of AgNPs (0, 1.5, 3, 6, and 12 mg/L of metallic silver).

As we showed in a previous paper [1], silver nanoparticles concentrations higher than 50 mg/L (3 mg/L of metallic silver) produce a very large amount of ROS that overwhelm the antioxidant system of the plant. The plants exposed to AgNPs concentrations of 100 and 200 mg/L (6 and 12 mg/L of metallic silver) showed an important decrease in number and length of roots, clearly appreciable by the naked eye as shown in Figure 3.

### 3.3. Cytotoxic and Genotoxic Effects

Morphological changes described above are consistent with the dose-dependent behavior observed on cell proliferation, reported as the mitotic index in Table 3. The most important decrease in the mitotic index was observed between the AgNPs concentrations of 100 and 200 mg/L (6 and 12 mg/L of metallic silver). These concentrations also are associated with the higher production of ROS and lipid peroxidation [1], in turn, responsible for the damage that leads to cell death. 

Similar dose-dependent response regarding the number and the length of shoots and roots was found after the exposure of sugarcane to these AgNPs at the same concentrations. Furthermore, the adverse effect was also attributed to ROS overproduction that overwhelms the antioxidant response of the plant [17].

Activation of antioxidant response by exposure of plants to metal nanoparticles (MNP) was used to promote positive effects on callus induction, shoot regeneration, and growth [18]. However, ROS overproduction has been identified as one of the main mechanisms by which MNP including AgNPs produce phytotoxicity [19].

Increased concentrations of ROS not only affect the cellular viability of exposed plants, but may also affect the integrity of their genetic material. Genotoxic effects of AgNPs in plants is scarcely studied, that is why we explore the genetic damage that could be produced by the exposure of *Vanilla planifolia* to several concentrations of AgNPs through the identification of nuclear aberrations shown as cells with bridges (CB), chromosomal fragments (CF), binucleated cells (BN), and micronuclei (MN).

The lower concentrations of silver nanoparticles (25 and 50 mg/L) do not generate significant damage in the genetic material neither as CB, CF, BN or MN compared with the control group, but an increase in the frequency of CB, CF, and BN were observed with the increment of silver nanoparticles concentrations without the presence of MN.

On the other hand, the higher concentrations of AgNPs, 100 and 200 mg/L (6 and 12 mg of metallic silver), administered to *Vanilla planifolia* continue with the observed tendency in the lower concentrations, increase aberrations in nuclear material as the concentration of AgNPs increase. The frequency of micronuclei registered was 1.5 and 3 with the exposure to 100 mg/L (6 mg/L of metallic silver) and 200 mg/L (12 mg/L of metallic silver), respectively.

**Figure 4:**
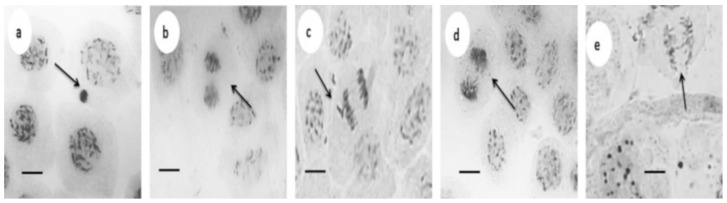
Chromosomal aberration and nuclear observed in root tips cells of *V. planifolia* at 200 mg L^−1^ of AgNPs after six weeks of in vitro culture. (**a**) Cell with micronucleus; (**b**) binucleated cell, (**c**) cell in anaphase with a chromosomal fragment, (**d**) cell in telophase with laggard, and (**e**) cell in anaphase with a bridge. Arrows indicate the produced damage in each case. Bar = 10 µm.

As far as we know, this is the first study that reports the genotoxicity on *Vanilla planifolia* Jacks using the micronuclei test. Figure 4 shows the chromosomal aberrations found in root tip cells of *Vanilla planifolia* exposed to AgNPs 200 mg/L (12 mg/L of metallic silver).

Due to the lack of comparison data for this plant, we summarize in Table 4 some of the genotoxic effects reported in the literature for different AgNPs formulations administered to several plants. The summary is not intended to be exhaustive but useful for assessing the significance of the results related with chromatic aberrations found in this work.

Table 4 shows that practically all studied formulations of AgNPs produce DNA damage in plants that have been exposed to these nanomaterials by different times and concentrations. In general, it could be established that a concentration- and time-dependent increase frequency of cells with chromatic aberrations (CA) and micronuclei (MN) was observed in plants exposed to AgNPs, independently of the treated plant or the AgNPs formulation employed.

The highest exposure time employed for the different AgNPs formulations compiled in Table 4, without including our results, is 10 days [24]. In all examples, chromatic aberrations or micronuclei appears with the highest concentrations and the effect increases over time, except for AgNPs-citrate formulation that after 72 h and 100 µM of silver administered, do not show differences with the untreated control plants [26]. The same concentration-dependent behavior regarding chromatic aberrations induction was found in our study when plantlets of *Vanilla planifolia* were exposed to several similar concentrations of our AgNPs formulation but with a 14-fold higher exposure time. (42 days = 1008 h).

Only one other report compiled in Table 4, besides ours, quantified silver content in their AgNP formulations [26]. The authors of this work evaluated the genotoxic damage induced by different formulations of AgNPs in the reference system *Allium cepa* [30], with similar concentrations than that we use in *Vanilla planifolia*. In this study, they found differences in the physiological and biochemical indicators such as ROS concentration, lipid peroxidation, and antioxidant response, mainly related with the size and coating of the three AgNPs formulations evaluated, AgNPs-citrate, AgNPs-PVP and AgNPs-CTAB. (PVP: poly(vinylpyrrolidone); CTAB: Cetyl trimethylammonium bromide).

Although it has been established that cytotoxic and in turn, the genotoxic effects depend on the size, coating, and exposure time [9,18,31,32], we believe that content of silver is also a fundamental component that must be reported for each AgNPs formulation generated, since the metal is the main active component responsible of the effects produced in the biological systems. Thus, we consider that minimum characterization data of nanoparticles including size, the coating agent (if exist), exposure time and silver concentration of the stock suspension employed must be reported. This in order to systematize the evaluation of the cytotoxic and genotoxic effect produced by nanomaterials, in this particular case, AgNPs.

Once analyzed data compiled in Table 4, we can suggest that our AgNPs formulation despite producing a cytotoxic effect at doses of 100 and 200 mg/L (6 and 12 mg/L of metallic silver), does not produce an important damage that can be considered as genotoxic on *Vanilla planifolia* plantlets that have been exposed even to 200 mg/L of nanoparticles (12 mg/L of metallic silver) for a quite long exposure time, 42 days. This is proposed considering that, despite the presence of DNA damage such as cells with bridges, binucleated cells, or chromosomal fragments, these errors can be solved by the cells. Then, the parameter that finally defines the irreversible genotoxic effect is the presence of micronuclei. In this sense, the increase of micronucleus in these plantlets after the long exposure time and concentrations employed could be considered as minimal comparing the effect observed in other systems. However, since the lack of positive genotoxicity control, this is only a suggestion. In our knowledge, this is the first report where cytotoxic and genotoxic parameters were determined after a very long exposure period. Definitely, further analysis must be done to study the real range of micronuclei, if it is possible to establish, for *Vanilla planifolia* in normal conditions and identify the magnitude of the damage due to the presence of AgNPs.

As we previously established, this AgNPs formulation was extremely effective to eliminate bacterial contamination with a concentration of 25 and 50 mg/L through a temporary immersion system without affectation of the plantlet. Additionally, with this AgNPs concentration, a hormetic effect was observed triggered by the increase of plantlet antioxidant response and an improvement in the capture and use of the nutrients [1]. Furthermore, in this work was identified that this concentration (50 mg/L or 3 mg/L of metallic silver) did not hinder shoot and root growth, without an important decrease on the mitotic index and with the absence of genotoxicity, all this after a very long-term exposure to AgNPs.

It is possible that promotion of growth through oxidative stress generation also promotes somaclonal variation due to the adaptation to the new in vitro growth conditions, which can be useful for increasing genetic variability of *Vanilla planifolia* crops, as was found for other systems, such as *Anthurium* [33] and apple [34], among others. Genetic improvement is not only important to satisfy raw material demands, but could also be useful for the preservation of the decimated wild population.

As far as we know, only three works reported the effect of AgNPs with respect to the induction of polymorphism or at the level of protein expression due to modifications in the genome with very different results [14,27,28]. In all systems, positive effects on plants were observed when exposed to low concentrations of AgNPs. However, is not possible to compare directly because the metallic silver content was not reported. On *Triticum aestivum* L. cv. Blasco no affectation was observed with the addition of 32 mL of 10 mg/L of AgNPs solution for 4 h [14]. A decrease in the genome template stability (GTS) was observed on *Solanum lycopersicum L*. treated with 10 mg/L or more of AgNPs after 336 h of exposure [28], which could improve the genetic variability of the species. Finally, lower expression of 1-aminocyclopropane-1-carboxylate synthase (ACS)—a key enzyme in ethylene biosynthesis—was observed on *Tecomella undulata* (Roxb.) Seem. with 30 mg/L after 384 h [27]. 

### 3.4. Somaclonal Variation

**Figure 5:**
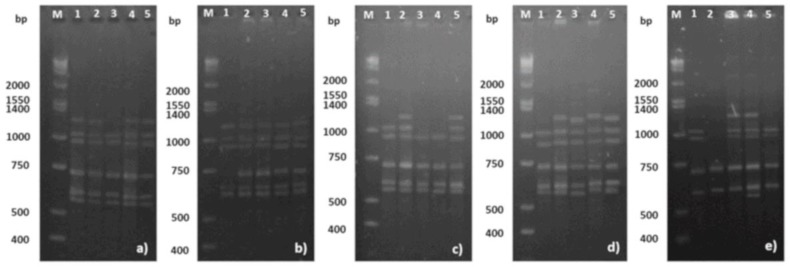
Electrophoresis pattern of ISSR banding profiles of five plants (1–5) of *V. planifolia* exposed to AgNPs for six weeks on in vitro culture. The amplification for UBC 825 primer corresponding to (**a**–**e**) 0, 25, 50, 100, and 200 mg/L of AgNPs, respectively. M = molecular mass marker 1 kbp plus DNA ladder; bp = base pairs.

To know the capability of AgNPs formulation studied in the present work to induce somaclonal variation on *Vanilla planifolia* plantlets, an inter-simple sequence repeat (ISSR) analysis was performed. ISSR is a widely used method to identify genetic diversity in plants through changes in repeat units of the genome [13,35]. The analysis of the banding profiles (Table 1, Figure 5 and Figures S1–S8) revealed the existence of polymorphism after exposure to several concentrations of AgNPs evaluated in leaves collected from five different plants. A total of 72 fragments from the selected ISSR markers compiled in Table 1 were amplified. Figure 5 shows the band pattern amplified with UBC (University of British Columbia) primer UBC-825. The band pattern of the others primers can be consulted in the Supplementary information (Figures S1–S8).

Exposure to AgNPs increases the polymorphism of *Vanilla planifolia* plantlets. Somaclonal variation was found in all treatments, showing a dose-dependent behavior. Plantlets without exposure to AgNPs showed a polymorphism of 15.28%. The polymorphism increases as AgNPs concentration does. The percentage of polymorphism observed was 18.06, 20.83, 23.61, and 25% for plantlets exposed to 25, 50, 100, and 200 mg/L of AgNPs for six weeks, respectively. The polymorphism found in this work is wide lower than that reported by Divakaran and Ramírez-Mosqueda in the range of 71–76% [36,37] by indirect organogenesis without additional stimulus. This could be attributable to the adaptation response of plantlets to imposed regeneration conditions, with or without oxidative stress by the presence or absence of AgNPs. 

*Vanilla planifolia* is considered at risk and is under special protection by the Mexican Government (Mexico City, Mexico) (NOM-059-SEMARNAT-2010) [38]. Thus, these results could represent a new alternative for the optimization of protocols that using concentrations of AgNPs ≤ 50 mg/L did not hinder the growth of *Vanilla planifolia* plantlets while inducing polymorphism, but without affectations in the mitotic index and with the absence of genotoxicity. 

## 4. Conclusions

In our knowledge, this is the first time that cytotoxic and genotoxic effect of a silver nanoparticles formulation has been studied on *Vanilla planifolia* plantlets. In addition, it is also the first report in which continuous exposure to silver nanoparticles is so long—Six weeks. Safe concentrations of this formulation, 25 and 50 mg/L, were identified. At these concentrations, a small decrease in the mitotic index, from 88 to 82%, and an increase in the frequency of cells with chromatic aberration, but without micronuclei, were observed. This damage could be considered negligible due to represents less than 1% of the total aberrations observed in 3000 cells. Even at the highest concentration (200 mg/L), damage of genetic material is minimum considering very long exposure to AgNPs (six weeks) and the time- and concentration-dependence behavior observed for other AgNP formulations. Finally, AgNPs’ safe concentrations promote the increase of polymorphism percentage, quite necessary to increase the genetic variability of this species considered at risk and under special protection. This work could represent a very important nanotechnological tool in the finding of alternatives to obtain large-scale and contaminant-free crops fundamental for several industries and, in this case, for the conservation of the species.

## Figures and Tables

**Table 1:** ISSR primers used for detecting somaclonal variation in *V. planifolia*.

Primer	Sequence (5′–3′)	°Tm (°C) ^2^	No. of Bands	Range (bp) ^3^	Polymorphism (%)
UBC 809	AGAGAGAGAGAGAGAGG	45	10	300–2000	30
T 06	AGAGAGAGAGAGAGAGGT	50	9	300–1550	66.66
UBC 840	GAGAGAGAGAGAGAGAYT ^1^	50	8	200–1400	25
UBC 836	AGAGAGAGAGAGAGAGYA ^1^	50	7	200–1550	42.86
UBC 812	GAGAGAGAGAGAGAGAA	50	7	200–1400	28.57
UBC 825	ACACACACACACACACT	51	6	500–3000	83.33
UBC 808	AGAGAGAGAGAGAGAGC	52	10	200–750	60
T 05	CGTTGTGTGTGTGTGTGT	54	8	300–2000	25
C 07	GAGAGAGAGAGAGAGAC	56	7	300–1400	71.43

^1^ Y″: C or T residues. ^2^ °Tm: annealing temperature. ^3^ bp: base pair.

**Table 2:** Physicochemical characteristics of silver nanoparticles commercial batch used in this work.

Properties	Mean
Metallic silver content	12 mg/L
Form Factor (Spheroid)	0.82
Roundness	0.88
Size interval of metallic silver particles by TEM (nm)	1–80
Average diameter by TEM (nm)	38 ± 15
Zeta potential (mV)	−15
Surface plasmon resonance (nm)	420

**Table 3:** Cytotoxic and Genotoxic effect of AgNPs on *V. planifolia* growth in vitro for six weeks.

AgNPs (mg L^−1^) ^1^	Cells in Division	Mitotic Index (%)	Aberration (%) ^2^				Total Aberration (%)
			CB	CF	BC	MN	
0	2582 ± 92 *	88.21 ± 0.48 *	0.03	0	0	0	0.03 ± 0.00 *
25 (1.5)	2348 ± 75 *	83.18 ± 1.16 *	0.10	0.08	0	0	0.18 ± 0.06 *
50 (3.0)	2338 ± 87 *	82.15 ± 2.40 *	0.30	0.50	0.17	0	0.97 ± 0.03 **
100 (6.0)	1786 ± 99 **	60.35 ± 0.90 **	1.5	1.0	0.16	1.5	4.16 ± 0.17 ***
200 (12.0)	1018 ± 72 ***	33.53 ± 1.91 ***	1.5	1.4	1.0	3.0	6.90 ± 0.22 ****

^1^ Values in brackets correspond to the metallic silver content in AgNPs formulation. ^2^ The total number of cells counted was 3000. CB: Cells with bridges; CF: Chromosomal fragments; BC: Binucleated cells; MN: Micronuclei. Average values ± standard error within a column followed by the same number of asterisks are not significantly different according to Tukey’s test at *p* ≤ 0.05.

**Table 4:** DNA damage and/or genotoxic effects observed in diverse plants exposed to several exposure times and concentrations of different AgNPs formulations.

Plant	AgNPs Source and Physicochemical Properties	Active Component Concentration (Metallic Silver Content)	Exposure Time and (AgNPs) Used	DNA Damage or Genotoxic Effect	Ref.
*Vanilla planifolia*	Commercial Vector-Vita PVP-AgNPs Size: 35 ± 15 nm, coating agent: PVP; ζ potential: −15 mV; hydrodynamic diameter: 70 nm	Metallic silver content quantified by ICP-OES 1.5, 3, 6, and 12 mg/L of metallic silver 13.9, 27.8, 55.6, and 111.25 µM of metallic silver	42 days (1008 h) 25, 50, 100, and 200 mg/L of AgNPs	A dose-dependent increase in the frequency of cells with CA. 1.5 and 3 MN were observed in 3000 counted cells for the concentrations 100 and 200 mg/L, respectively	This work
*Allium cepa*	Commercial Sigma-Aldrich size: <100 nm, purity: 99.5% trace metal basis, coating agent: NR	NR	4 h 25, 50, 75, and 100 mg/L	CA and cell disintegration.	[20]
*Vicia faba*	Commercial Ocean Nanotech LLC, size: 60 nm; purity: 99.5% trace metal basis, coating agent: NR Characterization made by the authors Size: 63 ± 41 nm, ζ potential: −33.2 mV	NR	4 h of exposure and 24 h of recovery 12.5, 25, 50, and 100 mg/L	Dose-dependence increase in the frequency of cells with CA and MN. MN frequency with 100 mg/L of AgNPs is triplicated compared with control (control 5.86 ± 0.66; AgNPs 100 mg/L: 18.4 ± 0.75).	[21]
*Nicotiana tabacum*	Commercial Sigma-Aldrich Size: <100 nm, purity: 99.5% trace metal basis, coating agent: NR Characterization made by the authors size: TEM 70–130 nm, av. ~125 nm; SEM: 90–180 nm, av. 120 nm; ζ potential: −4.86 mV	NR	24 h 25, 50, and 75 mg/L	No damage was observed in nuclei isolated from shoots. Nuclei isolated from roots exposed to 50 and 75 µg/mL shown DNA damage determined by comet assay. Dose-dependence for DNA damage.	[22]
*Triticum durum Desf.* cv. *Beni Sweif 1*	Synthesis, spherical, size: ~20 nm; coating agent: NR	NR	Soaked by 24 h in AgNPs solution and germinated by a period of 72 and 120 h, respectively. No concentrations reported	Time-dependent increase in the CA and MN frequency	[23]
*Hordeum vulgare* L. cv. *Giza 130*	Synthesis, spherical, size: ~20 nm; coating agent: NR	NR	Soaked by 24 h in AgNPs solution and germinated by a period of 72 and 120 h, respectively. No concentrations reported	Time-dependent increase in the CA and MN frequency	[23]
*Pithophora oedogonia (Mont.) Wittrock/Chara vulgaris Linn.*	Synthesis; size: 10–15 nm, coating agent: NR	NR	5 and 10 days 0.9 and 1.5 mM	CA observed with 0.9 mM after exposure of 5 days. Longer exposure (10 days) or higher concentrations enhance the magnitude of CA.	[24]
*Triticum aestivum* L.	Green synthesis: Rhodophyta extraction + AgNO_3_ Chemical synthesis: NaOH + AgNO_3_ + PEG No characterization data, coating agent: NR	NR	8, 16, and 24 h 10, 20, 40, and 50 mg/L	Both AgNPs showed concentration- and time-dependent increase in the frequency of cells with CA and MN.	[25]
*Triticum aestivum* L. cv. Blasco	Commercial nanoComposix Size: 10 nm, coating agent: PVP Characterization made by the authors Size: 13.2 nm	NR	Soaked by 4 h in 32 mL of 1 and 10 mg/L PVP-AgNPs solution, respectively.	No differences between the genetic polymorphism of roots treated with AgNPs and control samples by AFLP.	[14]
*Allium cepa*	Synthesis AgNPs-citrate, size: 61.2 ± 33.9 nm; TEM: rod-like; ζ potential: −39.8 ± 3.4 mV AgNPs-PVP, size: 9.4 ± 1.3 nm; TEM: spherical; ζ potential: −4.8 ± 0.6 mV AgNPs-CTAB, size: 5.6 ± 2.1 nm; TEM: spherical; ζ potential: 42.5 ± 2.7 mV	Metallic silver content quantified by ICP-MS for each sample 25, 50, 75, and 100 µM	72 h 25, 50, 75, and 100 µM	No DNA damage was observed with any of the AgNPs-citrate concentrations employed. Increase in tail DNA was recorded after exposure to AgNPs-PVP at 100 μM concentration. AgNPs-CTAB produces DNA damage only with 50 μM concentration.	[26]
*Tecomella undulata* (Roxb.) Seem.	NR	NR	16 days (384 h) 30, 60, and 120 mg/L	More than 30 mg/L of AgNPs decreases ACS expression levels	[27]
*Solanum lycopersicum* L.	Commercial Sigma-Aldrich (Catalog number 576832) Nanopowder, size: <100 nm, PVP as dispersant, purity: 99.5% trace metal basis	NR	14 days (336 h) 10, 20, 40, and 80 mg/L	GTS decreases as AgNPs concentration increases.	[28]
*Lathyrus sativus* L.	Synthesis. All have shown spherical shape AgNPsI: AgNO_3_ + extract. 14 ± 5.4 nm AgNPsII: AgNO_3_ + TSC+ extract. 19.2 ± 6.6 nm AgNPsIII: AgNO_3_ + TSC + PVPV + extract. 18.8 ± 6.6 nm AgNPsIV: AgNO_3_ + TSC + PVP + extract. 44.6 ± 13.2 nm	NR	Exposure for 3 h and recovery time of 4, 8, 12, and 24 h 1, 5, 10, 20, and 40 mg/L	Authors report that all AgNPs induce genotoxic effects from the concentration of 1 mg/L, with exception of AgNPsIV which induced genotoxicity only at the higher concentration of 40 mg/L.	[29]

NR: no reported; ICP-EOS: inductively coupled plasma optical emission spectrometry; ICP-MS: inductively coupled plasma mass spectrometry; PVP: poly(vinylpyrrolidone); PVPP: polyvinyl polypyrrolidone; TSC: trisodium citrate; CTAB: Cetyl trimethylammonium bromide; PEG: poly(ethylene glycol); MN: micronuclei; CA: chromosomic aberrations which include chromatin bridges, stickiness, disturbed metaphase, multiple chromosomal breaks. AFLP: Amplified fragment length polymorphism; ISSR: Inter-Simple Sequence Repeat; ACS: 1-aminocyclopropane-1-carboxylate synthase; GTS: Genome template stability.

## Supplementary Materials

The following are available online at http://www.mdpi.com/2079-4991/8/10/754/s1.
