# Supplementary material for: Cytotoxic, Genotoxic, and Polymorphism Effects on Vanilla planifolia Jacks ex Andrews after Long-Term Exposure to Argovit® Silver Nanoparticles
Source: Nanomaterials (Basel). 2018 Sep 25;8(10):754. doi: 10.3390/nano8100754 (PMC6215222; doi:10.3390/nano8100754)
Supplement: Supplementary file 1 [file nanomaterials-08-00754-s001.pdf]

Supplementary Material

Cytotoxic, Genotoxic, and Polymorphism Effects on *Vanilla planifolia* Jacks ex Andrews after Long-Term Exposure to Silver Nanoparticles

Jericó Jabín Bello-Bello [1,*], José Luis Spinoso-Castillo [2], Samantha Arano-Avalos [2], Eduardo Martínez-Estrada [2], María Evarista Arellano- García [3], Alexey Pestryakov [4], Yanis Toledano-Magaña [5], Juan Carlos García-Ramos [5,*] and Nina Bogdanchikova [6]

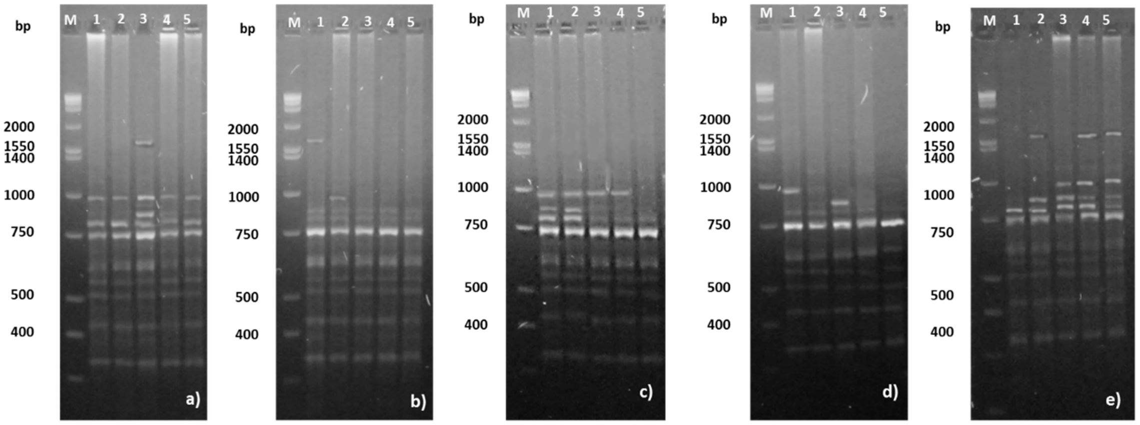

Figure S1. Electrophoresis pattern of ISSR banding profiles of five plants (1–5) of *V. planifolia* exposed to AgNP for six weeks on in vitro culture. The amplification for primer UBC 809 is shown. From left to right: 0, 25, 50, 100, and 200 mg/L of AgNPs, respectively. M = molecular mass marker 1 kbp plus DNA ladder; bp = base pairs.

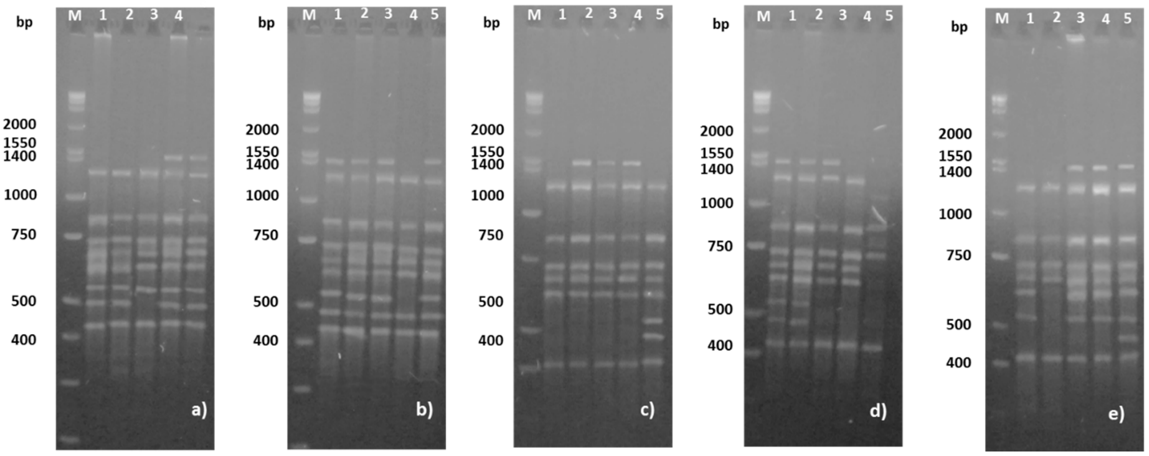

Figure S2. Electrophoresis pattern of ISSR banding profiles of five plants (1–5) of *V. planifolia* exposed to AgNP for six weeks on in vitro culture. The amplification for primer T06 is shown. From left to right: 0, 25, 50, 100, and 200 mg/L of AgNPs, respectively. M = molecular mass marker 1 kbp plus DNA ladder; bp = base pairs.

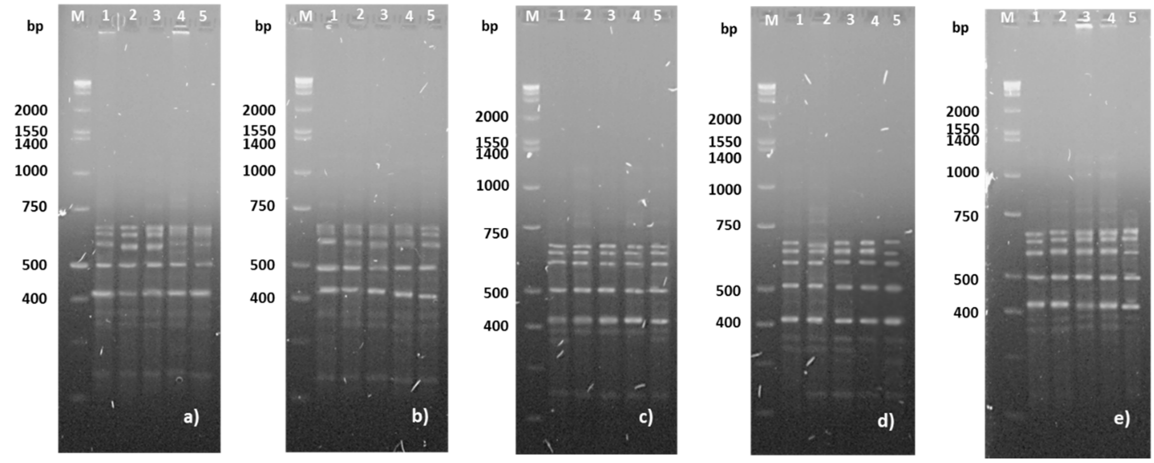

Figure S3. Electrophoresis pattern of ISSR banding profiles of five plants (1–5) of *V. planifolia* exposed to AgNP for six weeks on in vitro culture. The amplification for primer UBC 840 is shown. From left to right: 0, 25, 50, 100, and 200 mg/L of AgNPs, respectively. M = molecular mass marker 1 kbp plus DNA ladder; bp = base pairs.

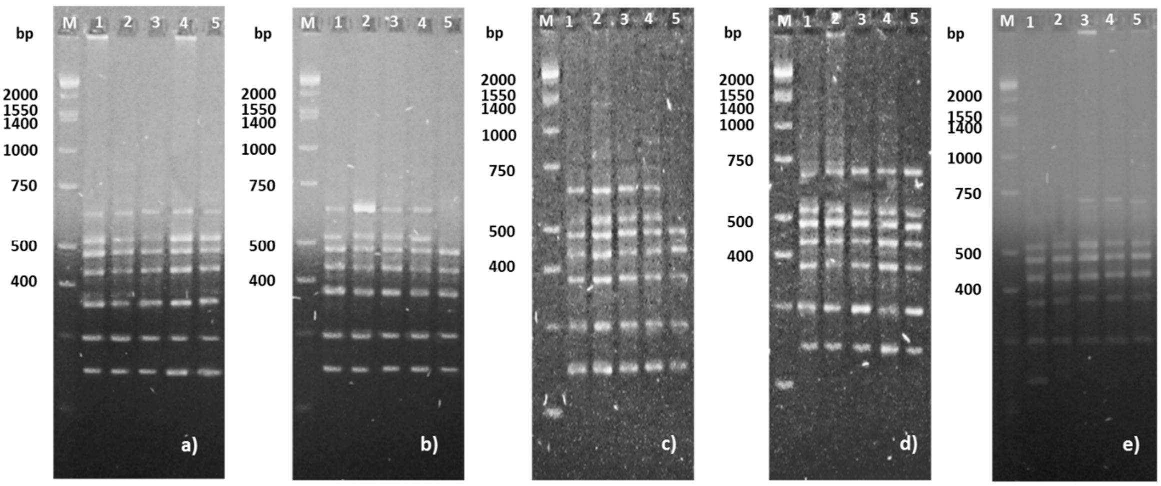

Figure S4. Electrophoresis pattern of ISSR banding profiles of five plants (1–5) of *V. planifolia* exposed to AgNP for six weeks on in vitro culture. The amplification for primer UBC 836 is shown. From left to right: 0, 25, 50, 100, and 200 mg/L of AgNPs, respectively. M = molecular mass marker 1 kbp plus DNA ladder; bp = base pairs.

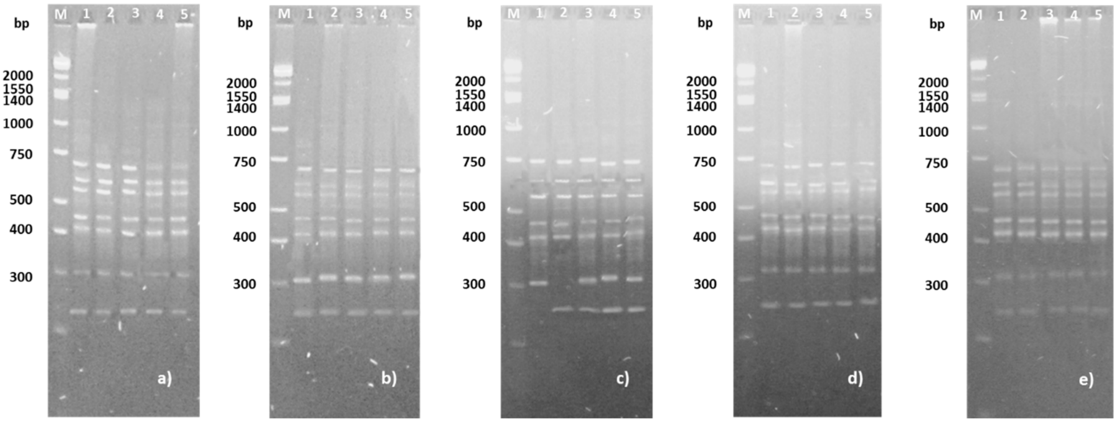

Figure S5. Electrophoresis pattern of ISSR banding profiles of five plants (1–5) of *V. planifolia* exposed to AgNP for six weeks on in vitro culture. The amplification for primer UBC 812 is shown. From left to right: 0, 25, 50, 100, and 200 mg/L of AgNPs, respectively. M = molecular mass marker 1 kbp plus DNA ladder; bp = base pairs.

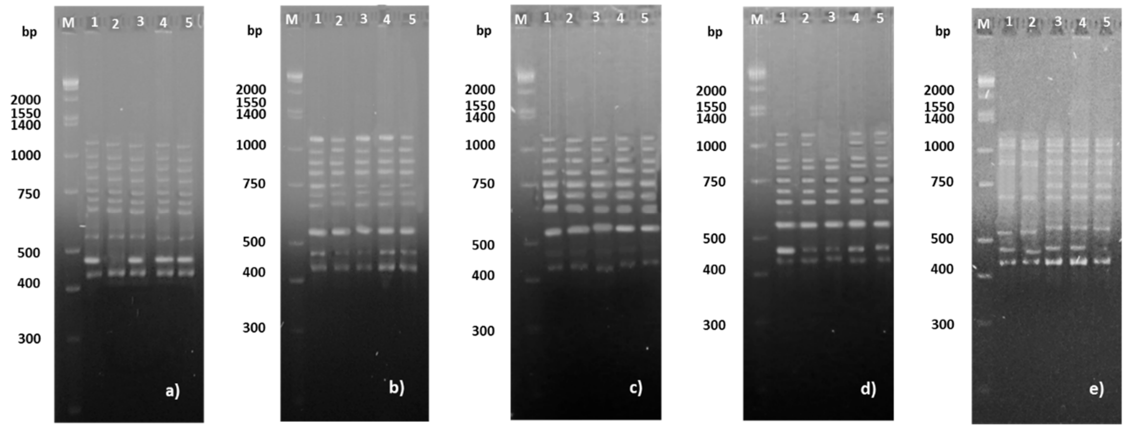

Figure S6. Electrophoresis pattern of ISSR banding profiles of five plants (1–5) of *V. planifolia* exposed to AgNP for six weeks on in vitro culture. The amplification for primer 808 is shown. From left to right: 0, 25, 50, 100, and 200 mg/L of AgNPs, respectively. M = molecular mass marker 1 kbp plus DNA ladder; bp = base pairs.

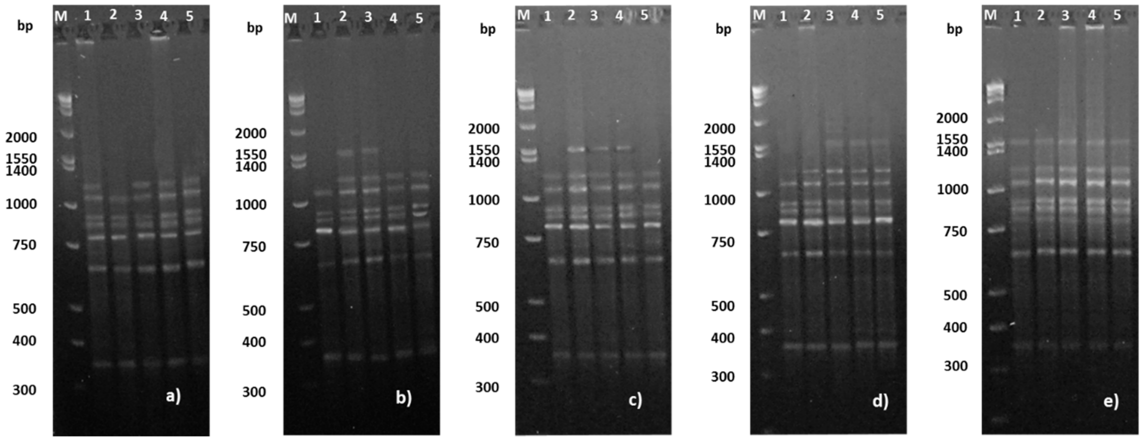

Figure S7. Electrophoresis pattern of ISSR banding profiles of five plants (1–5) of *V. planifolia* exposed to AgNP for six weeks on in vitro culture. The amplification for primer T05 is shown. From left to right: 0, 25, 50, 100, and 200 mg/L of AgNPs, respectively. M = molecular mass marker 1 kbp plus DNA ladder; bp = base pairs.

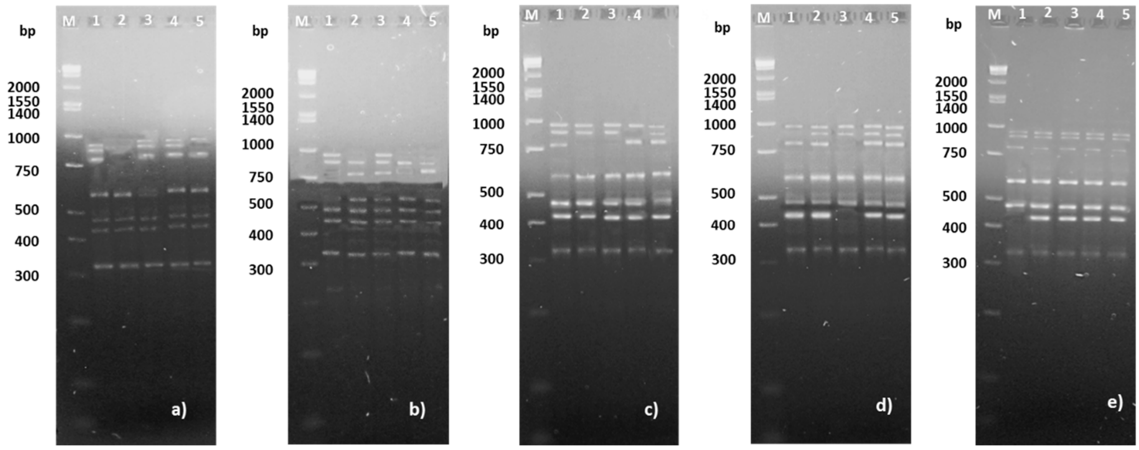

Figure S8. Electrophoresis pattern of ISSR banding profiles of five plants (1–5) of *V. planifolia* exposed to AgNP for six weeks on *in vitro* culture. The amplification for primer C07 is shown. From left to right: 0, 25, 50, 100, and 200 mg/L of AgNPs, respectively. M = molecular mass marker 1 kbp plus DNA ladder; bp = base pairs.
